# Zika virus infection during pregnancy protects against secondary infection in the absence of CD8^+^ cells

**DOI:** 10.1016/j.virol.2021.03.019

**Published:** 2021-07

**Authors:** Blake Schouest, Brandon J. Beddingfield, Margaret H. Gilbert, Rudolf P. Bohm, Faith Schiro, Pyone P. Aye, Antonito T. Panganiban, Diogo M. Magnani, Nicholas J. Maness

**Affiliations:** a Tulane National Primate Research Center, Tulane University, Covington, LA, USA; b Biomedical Sciences Training Program, Tulane University School of Medicine, New Orleans, LA, USA; c Department of Microbiology and Immunology, Tulane University School of Medicine, New Orleans, LA, USA; d Department of Medicine, University of Massachusetts, Boston, MA, USA

**Keywords:** Zika virus, Pregnancy, Nonhuman primates, Macaques, CD8^+^ T cells

## Abstract

While T cell immunity is an important component of the immune response to Zika virus (ZIKV) infection generally, the efficacy of these responses during pregnancy remains unknown. Here, we tested the capacity of CD8 lymphocytes to protect from secondary challenge in four macaques, two of which were depleted of CD8^+^ cells prior to rechallenge with a heterologous ZIKV isolate. The initial challenge during pregnancy produced transcriptional signatures suggesting complex patterns of immune modulation as well as neutralizing antibodies that persisted until rechallenge, which all animals efficiently controlled, demonstrating that the primary infection conferred adequate protection. The secondary challenge promoted activation of innate and adaptive immune cells, possibly suggesting a brief period of infection prior to clearance. These data confirm that ZIKV infection during pregnancy induces sufficient immunity to protect from a secondary challenge and suggest that this protection is not dependent on CD8 T cells.

## Highlights

• Zika infection during pregnancy provides protection from secondary infection.
• CD8 T cell responses are not needed for protection from secondary infection.
• Zika infection during pregnancy results in immunomodulatory transcriptional signatures but little IFN response.

## 1 Introduction

ZIKV was first isolated nearly seventy years prior to the Brazilian outbreak of 2015 (Dick et al., 1952; Zanluca et al., 2015), but the recent epidemic became associated with vertical transmission dynamics and congenital syndromes that were unprecedented for ZIKV or any other flavivirus (Plourde and Bloch, 2016). Although infrequent neurological manifestations, including Guillain-Barre syndrome, meningitis, and meningoencephalitis, became linked to infection in adults (Araujo et al., 2016; Avelino-Silva and Martin, 2016; Brasil et al., 2016; Ellul et al., 2016), the most severe neurological consequences were documented in infants born to mothers infected during pregnancy (Barton and Salvadori, 2016; Melo et al., 2016). Referred to as congenital Zika syndrome (CZS) (Moore et al., 2017), this collection of manifestations has provided the greatest justification to develop prophylactic and therapeutic countermeasures against the virus. Several murine and NHP models have been developed to understand mechanisms of maternal-to-fetal transmission and to develop and test antiviral therapies and vaccines (Aliota et al., 2016; Dudley et al., 2016; Hirsch et al., 2017; Koide et al., 2016; Magnani et al., 2018; Morrison and Diamond, 2017; Nguyen et al., 2017; O'Connor et al., 2018; Osuna et al., 2016), but NHPs may provide a superior model to study vertical transmission and congenital hazards due to the similarities in placental structure and gestational development to humans (Morrison and Diamond, 2017).

Experimental ZIKV vaccine efforts to date have been successful, with a number of candidate vaccines having advanced to clinical trials, but an underappreciated consideration in vaccine design is whether protective responses can be attained in the context of pregnancy. Complex interactions between sex hormones and the immune system make pregnant women more susceptible to severe outcomes associated with a host of infections (Kourtis et al., 2014), so an important question in ZIKV vaccine design is whether immunity induced during pregnancy is sufficient to prevent subsequent infections and if this protection extends to infants born to women infected during pregnancy.

A recent study showed that NHPs infected during pregnancy establish long-term immune responses that are sufficient to protect against secondary challenge (Moreno et al., 2019), a finding that is also true in macaques outside of pregnancy (Aliota et al., 2016). Similar to other flaviviruses, ZIKV infection results in rapid neutralizing antibody titers (Coffey et al., 2017), suggesting that humoral immunity may be the most important correlate of protection. However, ZIKV-specific T cell responses have been described in mice (Elong Ngono et al., 2017; Huang et al., 2017; Pardy et al., 2017), macaques (Dudley et al., 2016), and humans (Grifoni et al., 2017, 2018; Ricciardi et al., 2017; Xu et al., 2016), so cell-mediated immunity might also have role in protection from secondary infection. Evidence in mice suggests that memory CD8 T cells have a role in controlling infection, as shown by the adoptive transfer of ZIKV-specific memory CD8 cells to naïve mice which confer protection to ZIKV challenge (Elong Ngono et al., 2017). However, the role of CD8 cells in protection from rechallenge in a previously exposed animal remains unknown. Additionally, whether this protection extends to heterologous viruses is an important open question, since the mutagenic potential of RNA viruses may limit the efficacy of immune responses raised by either vaccination or natural infection. To address these questions, we used the rhesus macaque model to explore whether ZIKV infection during pregnancy induces sufficient immunity to protect from rechallenge with a heterologous ZIKV isolate, and we also asked whether CD8 lymphocytes are an important component of this protection.

## 2 Materials and methods

### 2.1 Primary challenge and viral load quantification

Primary ZIKV inoculations of the primary ZIKV isolate Rio-U1 were described previously (Magnani et al., 2018). The challenge virus was isolated in 2015 from the urine of a pregnant woman in Rio de Janeiro (Bonaldo et al., 2016). Briefly, four Indian rhesus macaques were initially challenged with ZIKV Rio-U1 at 10^4^ plaque forming units (PFU) during the 3rd trimester of pregnancy, resulting in serum viremia that peaked at 3 days post infection (dpi) with 5–6 logs of viral RNA/ml in plasma that cleared between 14 and 28 dpi. One animal, M09, had detectable virus in amniotic fluid just prior to full term fetal harvest, between 35 and 40 dpi. Two infants were sacrificed for tissue harvest, but no evidence of *in utero* infection was present. The other two infants were kept alive for a viral challenge and behavioral observation, as described previously (Maness et al., 2019).

### 2.2 RNA-sequencing and analysis

Total RNA was extracted from PBMC pellets at the indicated timepoints using the Zymo Quick-RNA Miniprep kit. RNA was purified using the Zymo RNA clean & concentrator-25 kit and quantitated using the Qubit RNA BR assay kit (Thermo Fisher). A beta release of the Collibri 3’ mRNA Library Prep Kit (Invitrogen) was used to prepare libraries, and sequencing was carried out at the Tulane NextGen sequencing core using an Illumina NextSeq instrument with 150 cycles.

Sequencing data were aligned and mapped to the rhesus macaque genome (Mmul_10 assembly) using STAR (Dobin et al., 2013) with default settings in gene quantification mode. Differentially expressed genes (DEGs) were calculated using DESeq2 (Love et al., 2014), and pathway analysis was carried out using gene set variation analysis (GSVA) (Hänzelmann et al., 2013), gene set enrichment analysis (GSEA) (Subramanian et al., 2005), ReactomePA (Yu and He, 2016), and Ingenuity Pathway Analysis (IPA) (Qiagen). For GSVA analysis, gene sets in the Reactome databank were used, and pairwise comparisons among conditions were carried out using limma (Ritchie et al., 2015). Gene sets were considered significantly differentially enriched at p < 0.05. For GSEA analysis, a false discovery rate (FDR) below 25% was used to identify gene sets in the Hallmarks collection that were significantly enriched at 3 or 7 dpi relative to pre-infection. For these analyses, a gene set permutation of 1000 was utilized. In IPA, the two transcriptionally responding animals at 3 dpi (M08 and M09) were used to identify signaling patterns at this timepoint, while all 3rd trimester animals were analyzed at 7 dpi. Volcano plots, heatmaps, and Venn diagrams were generated using the Enhanced Volcano, pheatmap, and Venn Diagram packages in R, respectively. For heatmaps of read count data, log2-transformed read counts of genes responsible for core enrichment of the indicated gene sets are plotted.

### 2.3 CD8 depletion and rechallenge

Approximately nine months after the initial challenge, after the animals had given birth, two of four dams were depleted of CD8α+ lymphocytes (primarily NK cells and CD8^+^ T cells) using the anti-CD8α MT807R1 antibody (Nonhuman Primate Reagent Resource, RRID:AB_2716320) with a standard four-dose regimen over 10 days. CD8 depletion commenced 14 days prior to rechallenge with a heterologous ZIKV strain. CD8^+^ cell counts in blood were monitored by FACS analysis, and animals were screened for adverse events after each administration and none were observed.

Secondary inoculations of the heterologous Puerto Rican isolate PRVABC-59 were carried out at the same dose (10^4^ PFU), and route (subcutaneous) as the primary challenge. PRVABC-59 and Rio-U1 are approximately 99.6% identical at the nucleotide level. Blood and cerebrospinal fluid (CSF) were drawn on days 0, 3, 5, 7, 14, and 28 post challenge. Viral RNA was isolated from serum and CSF using the Roche High Pure Viral RNA Kit followed by quantification as described previously (Magnani et al., 2018). Animals were euthanized 28 dpi (n = 2) or 30 dpi (n = 2) after secondary challenge.

### 2.4 Anti-ZIKV neutralizing antibody titers

VeroE6 cells (ATCC# CRL-1586) were maintained in DMEM with 10% FBS. Cells were plated in 12-well plates (Fisher Scientific # FB012928) to be 90–95% confluent on the day of experiment. Serum was diluted 1:2 from 1:100 to 1:51,200 in serum-free DMEM using non-binding 96-well dilution plates (Corning# 3879). Approximately 50 PFU of ZIKV Rio-U1 was mixed with the serum dilutions and allowed to incubate for 1 h at 37 °C and 5% CO_2_. After incubation, the mixtures were moved to 12-well plates containing VeroE6 cells, previously rinsed to remove residual FBS, and allowed to adsorb for 1 h at 37 °C and 5% CO_2_ with gentle rocking every 15 min. After adsorption was complete, inoculum was removed and the cells were overlayed with a mixture of 1.2% Avicel and MEM containing 2% FBS, 1% anti-anti, glutamine, NEAAs, sodium pyruvate and 2.2 g/L sodium bicarbonate.

Plates were fixed 5 days post infection with 10% formalin and stained with crystal violet for counting of plaques. % Neutralization was calculated as ((# plaques in the no serum well- # plaques in the treated well)/# plaques in the no serum well) *100. Neutralization curves were generated in Prism using a 4-parameter logistic regression constrained at 0 at the bottom and 100 at the top. An NT_80_ was calculated from the curve for each sample.

### 2.5 Flow cytometry

Cryopreserved PBMCs were thawed and labeled with the following antibodies: CD16 AL488, CD169 PE, CD28 PE-CF594, CD95 PCP-Cy5.5, CD3 PE-Cy7, CD8 Pacific Blue, CD14 BV605, HLA-DR BV650, CD69 BV711, NKG2A APC, and CD4 APC-H7, followed by fixation, permeabilization, and labeling with an antibody against Ki67 AL700. Flow cytometry data were collected on a BD LSR II instrument and analyzed using FlowJo v10.

## 3 Results

### 3.1 Transcriptome analysis following primary challenge

**Fig. 1:**
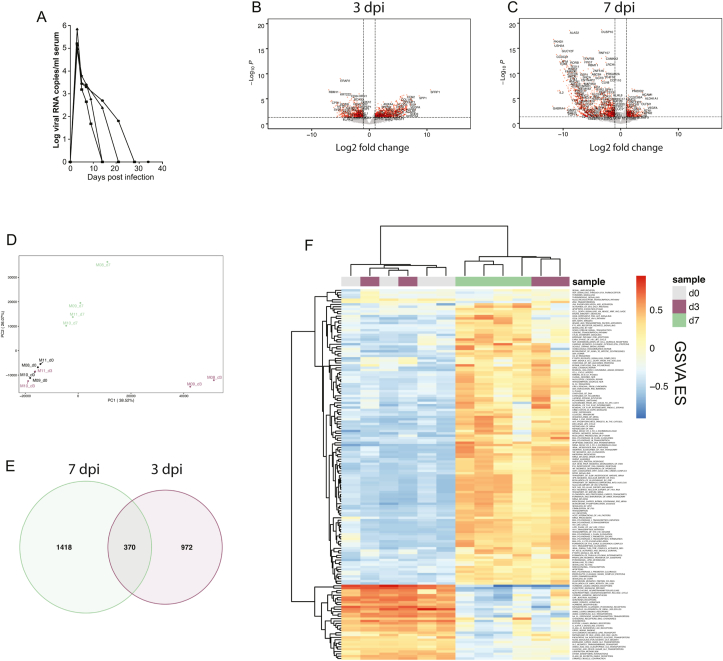
**Transcriptome analysis of primary infection during pregnancy**. (**A**) Serum viral loads following the primary challenge of four 3rd trimester macaques with ZIKV Rio-U1. (**B–C**) Volcano plots showing DEGs in PBMC at 3 dpi (B) and 7 dpi (C). (**D**) PCA plot showing the impacts of infection on the transcriptional landscape at 0, 3, and 7 dpi (d0, day 0; d3, day 3; d7, day 7; PC, principal component; consistent throughout). (**E**) Venn diagram showing the number of DEGs (p < 0.05) at 3 and 7 dpi. (**F**) Heatmap showing GSVA values among significantly modulated gene sets. Gene sets in the Reactome databank were included if statistical significance was attained in pairwise comparisons among timepoints. (ES, enrichment score.).

Following the primary challenge of four 3rd trimester rhesus macaques with ZIKV Rio-U1, all animals showed a rapid serum viremia which peaked at 5–6 logs viral RNA copies/ml at 3 dpi (Fig. 1a), as described previously (Magnani et al., 2018). To assess the quality of immune responses mounted during pregnancy, we carried out transcriptome analysis of PBMC at 3 and 7 dpi. Primary infection resulted in the up- and downregulation of many genes at both timepoints (Fig. 1b–c), but PCA revealed changes in gene expression at 3 dpi in only 2 of 4 animals (Fig. 1d). By 7 dpi, however, all animals showed more uniform responses (Fig. 1d) that were characterized by the differential expression of a greater number of genes (Fig. 1e). To identify how changes in gene expression affected global signaling patterns during infection, we carried out an unsupervised, phenotype-independent analysis by way of GSVA (Hänzelmann et al., 2013). Interestingly, the two transcriptionally responsive animals at 3 dpi appeared to show trends mirroring those seen in all animals at 7 dpi (Fig. 1f). At 7 dpi, the enriched gene sets generally related to inflammation, innate immunity, viral replication, cell cycle arrest, and cell death, while downregulated gene sets involved hormone signaling, neurotransmitter release, and small molecule transport (Fig. 1f).

**Fig. 2:**
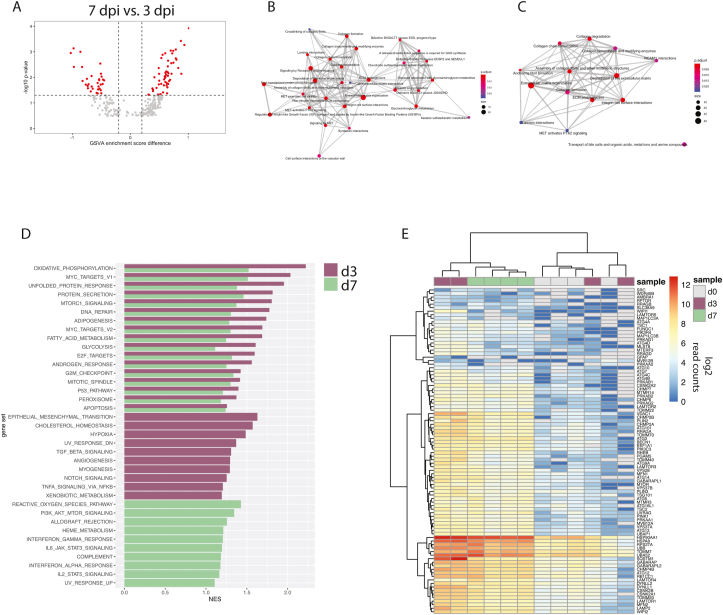
**Transcriptional signatures of infection during pregnancy**. (**A**) Volcano plot showing gene sets significantly modulated at 7 dpi relative to 3 dpi. (**B–C**) Enrichment map plots showing the interrelatedness of gene set networks at 3 dpi (B) and 7 dpi (C). (**D**) Gene sets from the Hallmarks collection that were significantly enriched (FDR<25%) by GSEA in 3rd trimester animals at both timepoints (*top*) or only at 3 dpi (*middle*) or 7 dpi (*bottom*). (**E**) Heatmap showing read count data for autophagy related genes.

Despite the up- and downmodulation of gene sets at 3 and 7 dpi (Figs. S1a–b) that in many cases appeared to be overlapping (Fig. 1f), a number of gene sets were differentially enriched at day 7 relative to day 3 (Fig. 2a), suggesting the possibility of divergent transcriptional signatures at these timepoints. Thus, we carried out a more detailed pathway analysis focusing on the progression of signaling patterns. Although there were fewer DEGs identified at 3 dpi relative to 7 dpi (Fig. 1e), a more varied functional fingerprint was evident at day 3 (Fig. 2b). Pathway analysis through GSEA showed that maintenance of structural proteins was affected at either timepoint, though diseases associated with metabolism and protein modification were detected only at 3 dpi (Fig. 2b–c). GSEA additionally revealed some level of immune activation at 3 dpi, with an effect on neutrophil degranulation and platelet activation (Fig. S1c). Interestingly, GAS6 was upregulated at 3 dpi (Fig. S1c), which is a bridging molecule that facilitates binding of ZIKV virions to the putative entry receptor AXL (Meertens et al., 2017). Gamma-carboxylation was also induced at this timepoint (Fig. S1c), a function important in the binding of GAS6 to AXL (Geng et al., 2017).

Viral infection is often associated with perturbations to metabolic processes, so to further characterize these effects which were initially identified by GSVA, we carried out targeted GSEA to compare phenotypes at 3 and 7 dpi to pre-infection. Indeed, we found that gene sets relating to cell respiration and lipid metabolism were among the most highly induced functions, and enrichment of these gene sets was generally greatest at 3 dpi (Fig. 2d). At day 3, there were additional signs of immunomodulation, including TGFβ signaling and angiogenesis, but by day 7, it was apparent that more of an inflammatory phenotype had emerged, marked by interferon (IFN) and proinflammatory cytokine signaling (Fig. 2d). The metabolic reprogramming at 3 dpi was characterized by changes in cell respiration (oxidative phosphorylation, glycolysis) and lipid metabolism (adipogenesis, fatty acid metabolism, cholesterol homeostasis, peroxisome) (Fig. 2d) that at the gene level showed activation in only the two early responding animals (Figs. S1d–e). Overarching effects on cell respiration and lipid metabolism might be explained by the induction of autophagy, which was also significantly enriched at 3 dpi (Fig. 2e). Autophagy has been shown to promote maternal-to-fetal transmission of ZIKV in mice through metabolic reprogramming in placental trophoblasts (Cao et al., 2017), and interestingly, one of the animals that showed early upregulation of autophagy signaling, M09 also had virus detectable in the amniotic fluid at multiple timepoints (Fig. S1f). M09 also had a higher peak serum viral load compared to the other 3rd trimester animals (Fig. 1b).

**Fig. 3:**
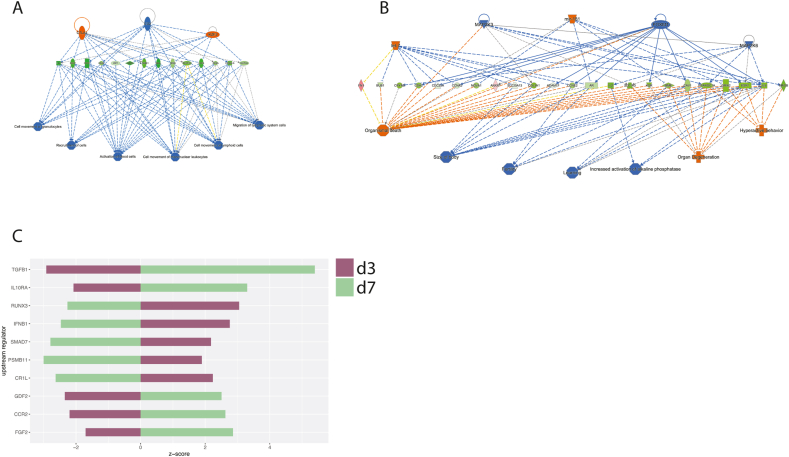
**Consequences of gene expression patterns in pregnant animals**. (**A-B**) Regulator effects pathways from IPA, showing the predicted activation states of upstream regulators and effects on canonical pathways at 3 dpi (A) and 7 dpi (B). (**C**) Predicted activation of biological regulators at 3 and 7 dpi. Z-score represents predicted activation of the molecule.

The 3rd trimester animals showed little evidence of IFN signaling especially at 3 dpi, although there was a generalized lack of IFN stimulated gene (ISG) expression at either timepoint (Fig. 1b–c). Rather, pathway analysis through IPA pointed to an immunomodulatory phenotype in the animals at 3 dpi that was characterized by depressed immune cell recruitment and activation (Fig. 3a). These patterns appeared to be driven by a decrease in a core set of chemokines (IL2) and chemokine receptors (CCR7, IL12RB1), together with downregulated adhesion proteins (SELP, CD48, SELL, ICOS, CD40LG), signaling molecules (IRF1, NFATC2), and activation markers (CD69, CD48) (Fig. 3a). By 7 dpi, there was downregulation of estrogen receptor (ESR1) and other genes relating to fertility (AR, CCNE2) and organ development (CAV1, PPARGC1A) (Fig. 3b). IPA predicted the up- and downregulation of several immunomodulatory molecules at both timepoints (Fig. 3c), including FGF2, which is known to support ZIKV infection by suppressing IFN signaling (Limonta et al., 2019). A number of other immune system modulators such as TGFβ, IL10, and type-I IFN were also inversely regulated between 3 and 7 dpi (Fig. 3c), suggesting a complex regulation of immunity over the course of infection.

### 3.2 CD8 lymphocyte depletion and rechallenge

**Fig. 4:**
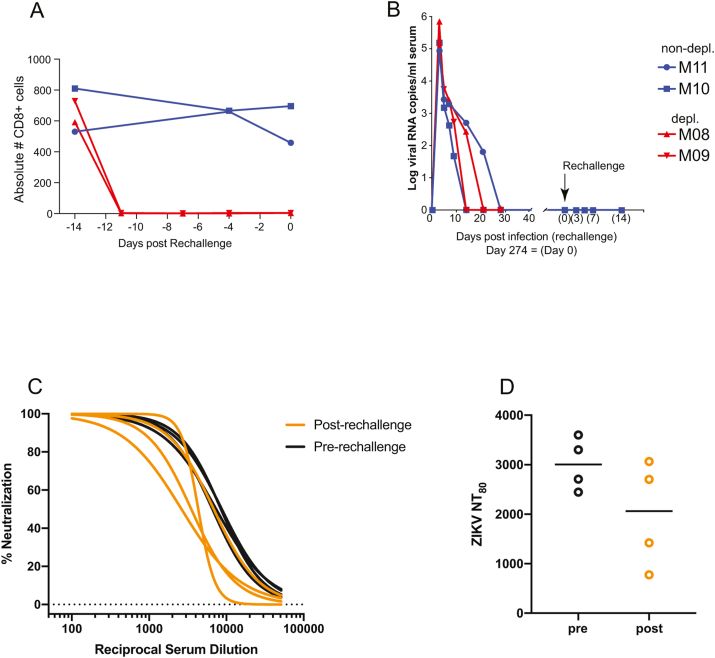
**CD8-depletion and rechallenge**. **A**) Absolute counts of CD8^+^ cells in the blood. CD8 T cells were depleted prior to rechallenge in two of four macaques that were initially infected during the 3rd trimester of pregnancy. (**B**) Serum viral loads following secondary challenge. The four macaques were rechallenged with the heterologous ZIKV strain PRVABC-59. (**C-D**) Plaque reduction neutralization test (PRNT) was used to assess anti-ZIKV neutralizing humoral immunity before and after rechallenge. Percent neutralization curves were calculated for all 4 animals at both time points (C). From the curves represented in C, we calculated the neutralization titer which resulted in an 80% reduction in plaques (NT80) (D).

To explore the role of CD8 T cells in protection from rechallenge, we carried out CD8 lymphocyte depletion, which resulted in a rapid decrease in CD8^+^ cell counts to an undetectable level (Fig. 4a). Given that ZIKV can persist in tissues long past the clearance of virus from the serum, viral loads were determined prior to rechallenge to ensure CD8 depletion did not result in recrudescent viremia from a cryptic reservoir, and none was detected (data not shown). Following rechallenge, viral RNA was not detected by qRT-PCR in any sample at any timepoint, suggesting complete immunity to the Puerto Rican strain (Fig. 4b).

### 3.3 Sustained anti-ZIKV neutralizing antibody titers persist until rechallenge

We used a classic plaque reduction neutralization test (PRNT) to quantify neutralizing antibodies in serum just prior to and 28 days following rechallenge. All animals harbored detectable neutralizing titers at the time of rechallenge (Fig. 4c), which appeared to decrease following rechallenge (Fig. 4d). However, this reduction was not significant and may have been due to assay-to-assay variability rather than an actual reduction.

### 3.4 Immune activation following rechallenge

We also assessed the activation of innate and adaptive immune cells as a surrogate of infection, given that viral RNA was not detected in the serum of any animal following rechallenge. Using a multicolor flow cytometry panel that we adapted from a previous ZIKV study (Schouest et al., 2019, preprint), we evaluated the proliferation (Ki67) and activation (CD69 or CD169) of T cells and monocyte subsets cells before and after rechallenge. CD169 is a biomarker of inflammation that has been used to track monocyte activation during acute ZIKV infection in macaques (Hirsch et al., 2017).

**Fig. 5:**
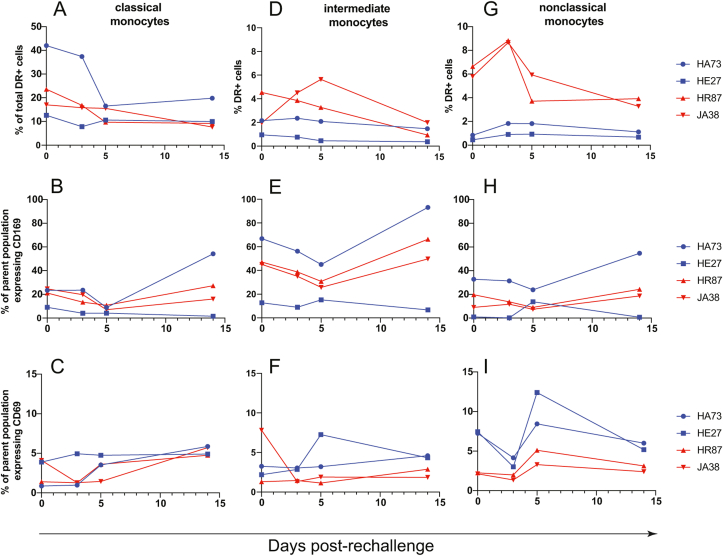
**Monocyte changes after ZIKV rechallenge**. Classical (CD14^+^, CD16^-^) (**A-C**), intermediate (CD14^+^, CD16^+^) (**D-F**), and non-classical (CD14^low/-^, CD16^+^) (**G-I**) monocytes were assessed for changes in frequency (A, D, G) and activation as measured by CD69 expression (B, E, H) and CD169 expression (C, F, I), after ZIKV rechallenge.

Classical and intermediate monocytes showed no discernible changes in frequency or activation following secondary challenge (Fig. 5a–f). However, nonclassical monocytes (CD14^-/low^, CD16^+^) expanded at 3 dpi in the CD8-depleted animals (Fig. 5g). Although nonclassical monocytes showed no change in CD169 expression (Fig. 5h), there was an increase in activation as measured by CD69 expression predominantly in the nondepleted animals at 5 dpi (Fig. 5i).

**Fig. 6:**
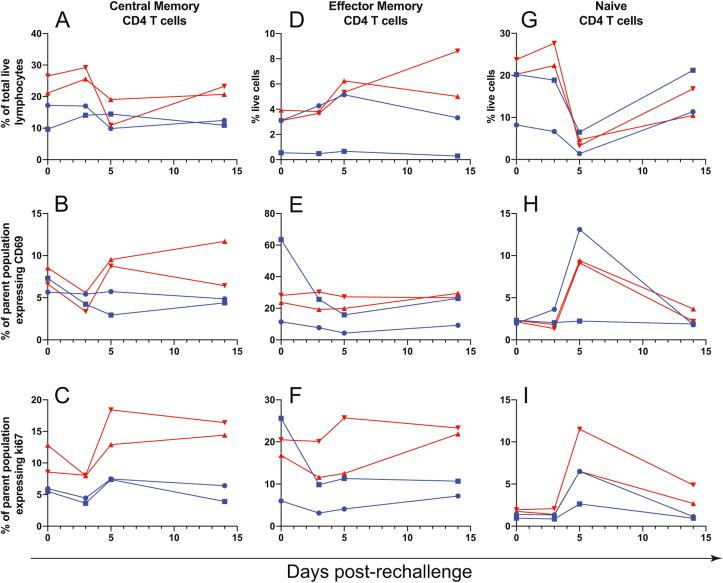
**CD4 T cell changes after ZIKV rechallenge**. Central memory (**A-C**), Effector memory (**D-F**), and naïve (**G-I**) CD4 T cells were assessed for changes in frequency (A, D, G), activation as measured by CD69 expression (B, E, H), and proliferation as measured by Ki67 expression (C, F, I), after ZIKV rechallenge.

Following rechallenge, central memory CD4 T cells expanded in frequency primarily in CD8-depleted animals (Fig. 6a), and these cells also showed increases in activation (CD69 expression, Fig. 6b) and proliferation (Ki67 expression, Fig. 6c) between 3 and 5 dpi in the same animals. Nondepleted animals showed an increase in central memory CD4 T cell proliferation during the same period (Fig. 6c), but the magnitude of this increase was less pronounced compared to the CD8-depleted animals. Effector memory CD4 T cells showed a modest increase in frequency in CD8-depleted animals at 3–5 dpi (Fig. 6d) without clear changes in activation or proliferation (Fig. 6e–f). Naïve CD4 T cells showed a striking drop in frequency in all animals between 3 and 5 dpi (Fig. 6g). The decline in naïve CD4 T cell frequency was concomitant with an increase in activation (CD69 expression, Fig. 6h) and proliferation (Ki67 expression, Fig. 6i) primarily in 3 of 4 animals.

**Fig. 7:**
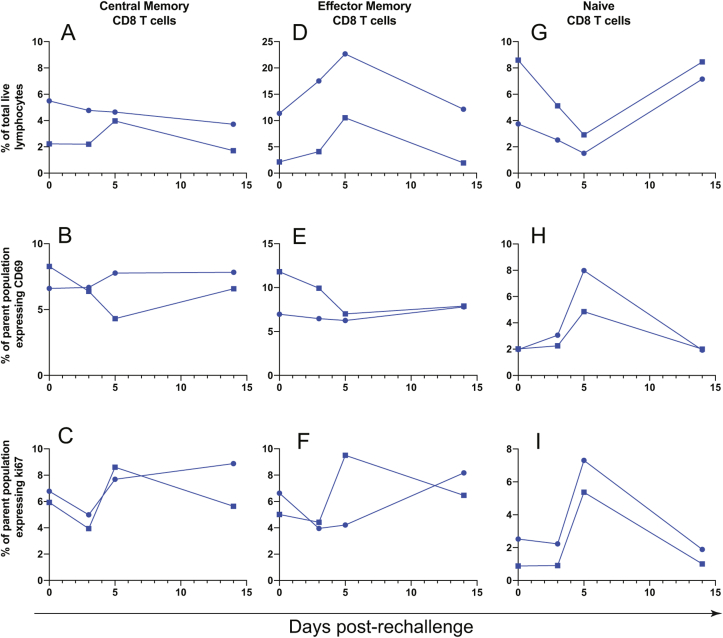
**CD8 T cell changes after ZIKV rechallenge**. Central memory (**A-C**), Effector memory (**D-F**), and naïve (**G-I**) CD8 T cells were assessed for changes in frequency (A, D, G), activation as measured by CD69 expression (B, E, H), and proliferation as measured by Ki67 expression (C, F, I), after ZIKV rechallenge.

Interestingly, phenotypic patterns in the CD8 T cell subsets of nondepleted animals generally mirrored those that occurred in the CD4 T cells of CD8-depleted animals. Although central memory CD8 T cells did not show appreciable changes in frequency (Fig. 7a) or activation (Fig. 7b) after rechallenge, there was a clear increase in proliferation between 3 and 5 dpi (Fig. 7c). Effector memory CD8 T cells expanded between the day of rechallenge and 5 dpi (Fig. 7d), but these cells did not become activated (Fig. 7e) and showed an increase in proliferation in only one of two nondepleted animals (Fig. 7f). In similar fashion to naïve CD4 T cells, naïve CD8 T cells dropped in frequency following rechallenge until 5 dpi (Fig. 7g) and showed marked increases in activation (Fig. 7h) and proliferation (Fig. 7i).

## 4 Discussion

ZIKV has been a known teratogen for some time, but the impacts of pregnancy on the quality of virus-specific immune responses have yet to be fully understood. The decrease in ZIKV incidence in the Western hemisphere since the peak of the outbreak in 2015 is of little reassurance until effective vaccines and therapeutics are mobilized. Although ZIKV vaccine candidates have performed well in preclinical settings (Abbink et al., 2018), the congenital risks associated with ZIKV infection introduce a set of challenges to vaccine development that requires special consideration and carefully chosen animal models. Pregnancy presents a substantially altered immunologic state to facilitate fetal development and protect the mother and developing fetus from infectious agents (Kourtis et al., 2014), so it follows that immune correlates of protection during pregnancy might differ from mechanisms that are important in nonpregnant individuals.

Whether immune responses induced during pregnancy, due to either infection or vaccination, are sufficient to protect from subsequent infection has begun to be examined in murine and NHP models. A recent study in IFN-deficient mice showed that a live-attenuated vaccine protected pregnant dams from infection and also prevented *in utero* transmission, and this protection appeared to be mostly dependent on neutralizing antibodies (Shan et al., 2019). The report cautioned that higher antibody titers were required to protect pregnant animals compared to nonpregnant animals, and pregnancy appeared to negatively impact the potency of the T cell response induced by the vaccine, which are important considerations in the evaluation of future vaccine candidates. A separate study in NHPs showed that animals initially challenged during pregnancy mount immune responses similar to nonpregnant animals, and these responses adequately protect against a secondary challenge (Moreno et al., 2019).

To identify signatures associated with ZIKV infection during pregnancy in macaques, we carried out transcriptome analysis of the blood of animals infected during the 3rd trimester, which revealed nuanced patterns of immune regulation that did not appear to involve IFN signaling. The absence of early IFN responses in these animals contrasts patterns of innate immunity that are known to limit ZIKV infection (Ngono and Shresta, 2018). The importance of the IFN response in the control of ZIKV is perhaps best illustrated in that mouse models often require IFN deficiency to replicate disease manifestations following infection, since ZIKV is unable to antagonize IFN in mice (Grant et al., 2016). In the context of pregnancy, evidence in mice indicates that IFN signaling confers protection from *trans*-placental infection (Miner et al., 2016), so the absence of IFN responses in the 3rd trimester macaques might have contributed to ZIKV crossing the placental barrier in one of the animals. The transcriptional landscape in these animals was instead characterized by a level of complexity that also entailed metabolic and hormonal effects that might have ultimately skewed immune outcomes. The suppression of immune cell homing and activation was particularly intriguing and might imply an immunomodulatory phenotype, possibly owing to maternal hormonal regulation. Although pregnancy has, in the past, been viewed as an immunosuppressive host-graft relationship to prevent fetal damage, current models recognize pregnancy as a progression of stages, each requiring unique immunological cues (Mor et al., 2017). In the pregnant animals, immune activation was altered even among the two timepoints we obtained post-challenge, possibly reflecting a complex regulation of immunity to ZIKV infection in the context of pregnancy. Although we also detected several gene expression signatures that were predicted to negatively impact reproductive function and organ development, it is not clear whether such transcriptional changes in PBMC would have an effect on the fetus. Additionally, any immunoregulatory phenotype that might have occurred ultimately did not compromise fetal health, as infants from two dams were born healthy and quickly cleared postnatal ZIKV challenge, and no adverse effects on nervous system development or behavior were noted, as described previously (Maness et al., 2019).

In addition to altered immune activation patterns, metabolic reprogramming through autophagy also appeared to characterize infection in these animals. Although autophagy generally aids in pathogen degradation and in the induction of immune responses during microbial infection (Ma et al., 2013), ZIKV and DENV, like other viruses, interact directly with autophagy pathways to promote replication (Chiramel and Best, 2018). Moreover, it has been shown in mice that ZIKV activates autophagy in placental trophoblasts to enhance vertical transmission (Cao et al., 2017), so it was fascinating that a pregnant macaque with evidence of early autophagy signaling patterns also had virus cross the placental barrier and a higher peak serum viremia at 3 dpi following the initial challenge. However, a separate animal that also showed induction of autophagy did not have elevated viremia compared to animals without autophagy signaling, so whether this signaling mechanism contributes to ZIKV replication in macaques requires further investigation. Since autophagy is at its core a degradative process that frees biomolecules such as lipids to enter energy producing pathways, functions we detected in pregnant ZIKV infected animals, metabolomic experiments may address whether autophagic flux is related to ZIKV infection generally or ZIKV infection during pregnancy specifically.

Although transcriptional patterns following the primary challenge showed that the animals failed to mount a robust IFN response, the lack of viremia following the secondary challenge suggests that the immune responses that did occur ultimately conferred complete protection. The presence of an anamnestic response in all 4 animals following the rechallenge prevents us from excluding the possibility of low-level viral replication that we failed to detect, but nonetheless, it is clear that immune responses mounted during pregnancy confer sufficient protection to reinfection in nonhuman primates. Such a finding is not entirely surprising, as several studies have shown an efficient generation of immunity by vaccines administered during pregnancy (Healy, 2012; Munoz et al., 2014; Ohfuji et al., 2011; Sperling et al., 2012). Outdated models portray pregnancy as a global suppression of immunity (Mor et al., 2017), but these perspectives are no longer generally accepted, as it has become clear that pregnancy is rather a complex alteration of particular immune subsets to balance fetal development and protection from infection (Kourtis et al., 2014). Indeed, pregnancy is a progressive biological process that requires a progressively adapting immune microenvironment (Mor et al., 2017).

Despite a small sample size that precluded statistical analysis, our data show phenotypic changes in immune cell populations that may indicate some level of cellular immune involvement in resistance to rechallenge. Nondepleted animals showed preferential expansion of effector memory CD8 T cells, while CD8-depleted animals showed greater increases in memory CD4 T cell subsets, possibly suggesting a compensatory CD4 response as we have observed previously in a cohort of male macaques that were similarly CD8 depleted prior to ZIKV challenge (Schouest et al., 2019). We caution that the two animals that were depleted of CD8 T cells prior to rechallenge were the same animals that showed early transcriptional activation patterns at 3 dpi, so we cannot rule out the possibility that animal variation influenced some of the phenotypic trends we observed here. Nonetheless, antibody-mediated depletion experiments in mice have also illustrated the redundancy of adaptive responses to ZIKV, with the depletion of individual immune cell populations resulting in alternative compensatory responses (Scott et al., 2018). Together, these studies begin to reveal the plasticity of immune responses to ZIKV that may coordinate to maintain overall immune integrity.

Although limited sample availability precluded analysis of antigen specific CD8^+^ T cell responses, the potential for CD8^+^ lymphocytes to aid in protection from rechallenge is intriguing. Vaccine induced CD8 T cells are important in protection from Ebola virus challenge (Gupta et al., 2005; Sullivan et al., 2011; Warfield et al., 2005), and the lack of CD8 T cell epitopes in the currently licensed DENV vaccine (Dengvaxia, Sanofi Pasteur) might contribute to some of the efficacy concerns associated with vaccination (Tian et al., 2019). Whether this experimental readout will also be true for ZIKV immunity in humans is unknown, but these findings nonetheless underscore the importance of CD8 responses in protection from these viruses generally and in vaccine design. ZIKV-specific CD8 T cells are described in multiple species (Elong Ngono et al., 2017; Grifoni et al., 2018; Huang et al., 2017; Pardy et al., 2017) and may be important for viral clearance in mouse models. CD8 T cells have active roles in controlling infections caused by other flaviviruses including West Nile virus (Grifoni et al., 2018; Klein et al., 2005; Shrestha and Diamond, 2004; Shrestha et al., 2006; Wang et al., 2003, 2006), DENV (de Alwis et al., 2016; Lam et al., 2017; Regla-Nava et al., 2018; Rivino and Lim, 2017; Shi et al., 2015; Yauch et al., 2009), and yellow fever virus (Akondy et al., 2009; Bassi et al., 2015; Co et al., 2009; Nogueira et al., 2013), which, given their relatedness, implies that CD8 T cells may be similarly important in limiting ZIKV infection.

Phenotypic changes among monocyte subsets were minimal, but monocytes are the primary targets of ZIKV in the blood (Foo et al., 2017; Michlmayr et al., 2017; O'Connor et al., 2018), so any alterations in frequency or activation of these cells are potentially interesting. The increases in nonclassical monocyte frequency and activation in CD8-depleted and nondepleted animals are intriguing because among monocytes, the nonclassical subset is preferentially targeted by ZIKV infection (O'Connor et al., 2018). Moreover, in pregnant women, Asian-lineage ZIKV infection selectively expands nonclassical monocytes and induces an M2-skewed immunosuppressive phenotype (Foo et al., 2017). Why nonclassical monocytes responded differently among CD8-depleted and nondepleted animals is unclear, but similar patterns occurred in a previous study from our group that also used CD8 depletion in a cohort of male rhesus macaques (Schouest et al., 2019). In that study, the collateral depletion of NK cells in CD8-depleted animals appeared to skew patterns of monocyte activation among treatment groups, which might have also been the case here. Together, these cellular immune data suggest some involvement of innate and adaptive cellular immune responses to the rechallenge virus, but the complete absence of viremia in both groups confirms that protection from rechallenge was robust and was likely due to the persistence of neutralizing antibodies induced by the primary challenge and maintained until rechallenge.

## 5 Conclusions

Together, our data confirm findings from a recent study in NHPs suggesting that pregnancy does not overtly impair immune responses to ZIKV infection, a finding with potential implications for vaccine design. We caution that the small sample size limited our ability to correlate immune patterns with pregnancy status, so the trends described here require verification. Additionally, as the cohort of animals used in immune phenotyping experiments was initially infected during the 3rd trimester of pregnancy, it remains possible that infection during the 1st or 2nd trimesters might produce less protective responses. Our findings add to a growing body of data describing the correlates of ZIKV-induced immunity in animal models of pregnancy, justifying vaccine research efforts in this unique subpopulation.

## Funding sources

This work was supported by a grant from the 10.13039/100000865Bill & Melinda Gates Foundation, Seattle, WA [OPP1152818 (Panganiban)]. The funders had no role in study design; in the collection, analysis and interpretation of data; in the writing of the report; or in the decision to submit the article for publication.

## CRediT authorship contribution statement

**Blake Schouest:** Methodology, Formal analysis, Investigation, Writing – original draft, Writing – review & editing, Visualization. **Brandon J. Beddingfield:** Methodology, Formal analysis, Investigation, Writing – review & editing. **Margaret H. Gilbert:** Methodology, Writing – review & editing, Supervision. **Rudolf P. Bohm:** Writing – review & editing, Supervision. **Faith Schiro:** Investigation, Writing – review & editing. **Pyone P. Aye:** Project administration. **Antonito T. Panganiban:** Conceptualization, Resources, Writing – review & editing, Funding acquisition. **Diogo M. Magnani:** Conceptualization, Methodology, Writing – original draft, Writing – review & editing. **Nicholas J. Maness:** Conceptualization, Methodology, Formal analysis, Resources, Writing – original draft, Writing – review & editing, Visualization, Supervision, Funding acquisition.

## Declaration of competing interest

The authors have no competing interests to declare.

## Appendix A Supplementary data

figs1**Pathway analysis of transcriptional signatures.** (**A-B**) Volcano plots showing gene sets significantly modulated at 3 dpi (A) and 7 dpi (B) relative to pre-infection. (**C**) Gene network showing signaling patterns and genes induced at 3 dpi. (**D-E**) Heatmaps showing read count data for genes relating to oxidative phosphorylation (D) and fatty acid metabolism (E). (**F**) Graph showing viral loads in the amniotic fluid (dotted line, open symbols) and serum (solid line, closed symbols) of M09 following primary challenge figs1 Pathway analysis of transcriptional signatures. (A-B) Volcano plots showing gene sets significantly modulated at 3 dpi (A) and 7 dpi (B) relative to pre-infection. (C) Gene network showing signaling patterns and genes induced at 3 dpi. (D-E) Heatmaps showing read count data for genes relating to oxidative phosphorylation (D) and fatty acid metabolism (E). (F) Graph showing viral loads in the amniotic fluid (dotted line, open symbols) and serum (solid line, closed symbols) of M09 following primary challenge
