# Supplementary figures and images for: Zika virus infection during pregnancy protects against secondary infection in the absence of CD8+ cells
Source: Virology. 2021 Jul;559:100–10. doi: 10.1016/j.virol.2021.03.019 (PMC8212702; doi:10.1016/j.virol.2021.03.019)

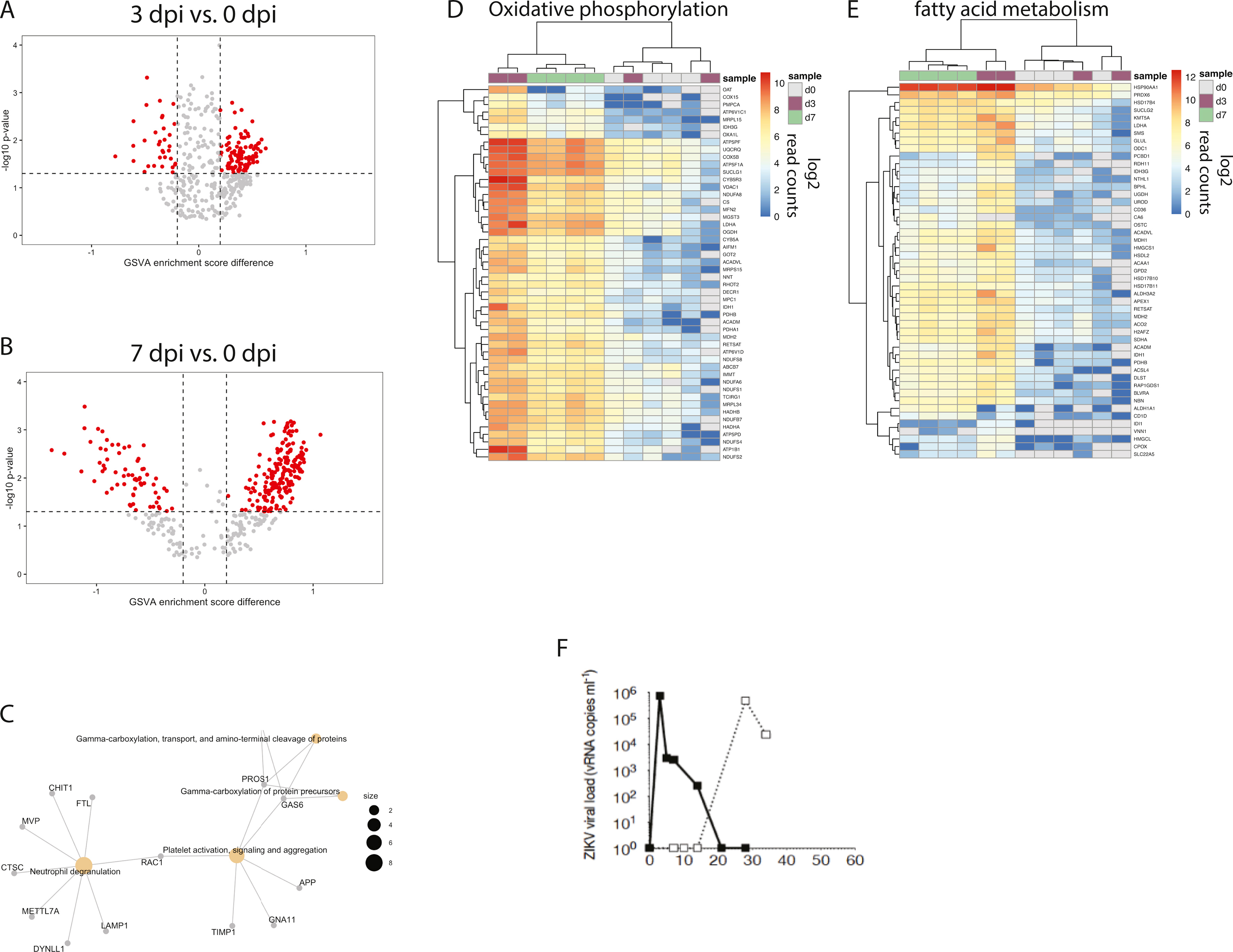

Supplement: figs1Pathway analysis of transcriptional signatures. (A-B) Volcano plots showing gene sets significantly modulated at 3 dpi (A) and 7 dpi (B) relative to pre-infection. (C) Gene network showing signaling patterns and genes induced at 3 dpi. (D-E) Heatmaps showing read count data for genes relating t — figs1 [file mmcfigs1.jpg]
